# Novel mutations in the ABCD1 gene caused adrenomyeloneuropathy in the Chinese population

**DOI:** 10.3389/fneur.2023.1126729

**Published:** 2023-02-28

**Authors:** Raoli He, Jian Zhang, Tianwen Huang, Guoen Cai, Zhangyu Zou, Qinyong Ye

**Affiliations:** ^1^Department of Neurology, Fujian Medical University Union Hospital, Fuzhou, Fujian, China; ^2^Institute of Clinical Neurology, Fujian Medical University, Fuzhou, Fujian, China; ^3^Fujian Key Laboratory of Molecular Neurology and Institute of Neuroscience, Fujian Medical University, Fuzhou, Fujian, China

**Keywords:** adrenomyeloneuropathy, X-linked adrenoleukodystrophy, spastic paraparesis, ABCD1, mutation

## Abstract

**Background:**

As a rare genetic disease, adrenomyeloneuropathy (AMN) is the most common adult phenotype of X-linked adrenoleukodystrophy (X-ALD). Mutations in the ABCD1 gene have been identified to cause AMN.

**Methods:**

We applied clinical evaluation, laboratory tests, and neuroimaging on three patients with progressive spastic paraparesis. In genetic analysis, we investigated ABCD1 gene mutations by whole-exome sequencing and Sanger sequencing. Bioinformatics tools were used to predict the effects of identified ABCD1 mutations on the protein.

**Results:**

All three patients were men with adult-onset disease, mainly characterized by progressive spastic paraparesis. Among them, two patients had peripheral neuropathy and one patient had signs of adrenal insufficiency. All three patients showed cerebral involvement on brain MRI, while two patients were found with diffuse cord atrophy on spinal MRI. High-VLCFA levels in plasma, as well as C24:0/C22:0 and C26:0/C22:0 ratios, were found in all three patients. In addition, three different ABCD1 mutations were identified in three unrelated Chinese families, including one known mutation (c.1415_1416delAG) and two novel mutations (c.217C>T and c.160_170delACGCAGGAGGC). Based on the clinical assessment, radiographic, biochemical, and genetic testing, the final diagnosis was AMN in these patients with spastic paraparesis.

**Conclusion:**

This study reported three patients with AMN and identified two novel mutations in the ABCD1 in the Chinese population. Our finding emphasized that X-ALD is an important cause of adult-onset spastic paraplegia. Thus, neuroimaging, VLCFA testing, and especially the detection of the ABCD1 gene have important implications for the etiological diagnosis of adult patients with spastic paraplegia.

## 1. Introduction

X-linked adrenoleukodystrophy (X-ALD, OMIM: 300100) is a rare progressive neurometabolic disorder affecting the adrenal cortex, testes, and myelin in the central nervous system. X-ALD is caused by mutations in the ABCD1 gene, which is a member of the ATP-binding cassette (ABC) transporter superfamily and encodes a peroxisomal membrane protein associated with very long-chain fatty acid (VLCFA) metabolism (1). Depending on the age of onset, the location of lesions, and the pace of progress, X-ALD is divided into several different phenotypes: childhood cerebral ALD, adolescent cerebral ALD, adult cerebral ALD, adrenomyeloneuropathy (AMN), cerebellar variant, Addison-only, asymptomatic, and presymptomatic female heterozygotes (2). Approximately 65% of patients develop AMN in adulthood, as it is the most common phenotype, which mainly manifests as slowly progressive gait disturbance and peripheral neuropathy (including large nerve fiber neuropathy and small nerve fiber neuropathy) (3, 4). In this study, we reported three Chinese pedigrees with AMN and identified two novel mutations in the ABCD1 gene.

## 2. Methods

### 2.1. Families and patients

The study was approved by the Ethics Committee of the Fujian Medical University Union Hospital. Written informed consent was obtained from all participants or their legal guardians.

Patients underwent a comprehensive clinical evaluation, including symptom evaluation, neurological examination (including cognitive function assessment), neuroimaging, neurophysiological testing, biochemical testing, and genetic testing. In particular, all participants underwent genetic testing only after informed consent was obtained from the patients or their legal guardians after comprehensive genetic counseling.

### 2.2. Neuroimaging

Magnetic resonance imaging (MRI) was used to detect pathological changes in the brain and the spine.

### 2.3. Biochemical testing

Plasma very long-chain fatty acids (VLCFAs) were assayed using gas chromatography—mass spectrometry. Plasma adrenocorticotropic hormone (ACTH) was measured by radioimmunoassay.

### 2.4. Genetic testing

We performed whole-exome sequencing (WES) on the probands of three pedigrees. Genomic DNA was extracted from whole blood samples using a QIAamp DNA Blood Mini Kit (Qiagen Inc., USA) following the manufacturer's protocol. The quantity and quality of obtained DNA samples were analyzed by using a NanoDrop 2000 spectrophotometer (Thermo Scientific, USA) and an Agilent 2100 bioanalyzer (Agilent Technologies, USA). Agilent SureSelect Human All Exon V6 (Agilent Technologies, USA) was used to enrich DNA fragments of human exons after the fragmentation of genomic DNA. Sequencing data were produced on the Illumina NovaSeq platform. The adaptor sequences were trimmed from the tail of sequencing reads using cutadapt (v1.15). Sequencing reads were aligned to the human reference genome (hg19) with BWA (v0.7.15). Duplicated reads were marked by Picard (v2.4.1). Qualimap (v2.2.1) was used to calculate base quality metrics, genome mapping rate, and the coverage of targeted regions. Base quality score recalibration, single nucleotide variants (SNVs), and small insertions or deletions (InDels) calling were performed following the best practice protocol of GATK (v3.8). Variant filtering was done by a finely tuned in-house script. Pass-filter variants were annotated using VEP (release 88). Subsequently, Sanger sequencing confirmed the presence of the mutation and segregation in three families.

### 2.4. Genetic and bioinformatics analysis

We first removed variants that met any of the following criteria: Population frequency in 1,000 Genome Project or gnomAD exome dataset (version 2.1) was larger than 0.01, and genotype was low confidence. The bioinformatics tools were used to predict the effects of the missense mutation on protein function: SIFT (5), PolyPhen2 (6), and MutationTaster (7). We then identified variants that fit the dominant and recessive inheritance models separately. The pathogenic evidence of candidate disease-causing variants was scored by InterVar (v1.0.8) (8). All the aforementioned analyses were performed on Seqmax (www.seqmax.com). On the contrary, we used SWISS-MODEL in order to predict the three-dimensional (3D) structure of the ABCD1 protein (swissmodel.expasy.org) to show the effect of identified mutations.

## 3. Results

### 3.1. Clinical characterization

**Figure 1:**
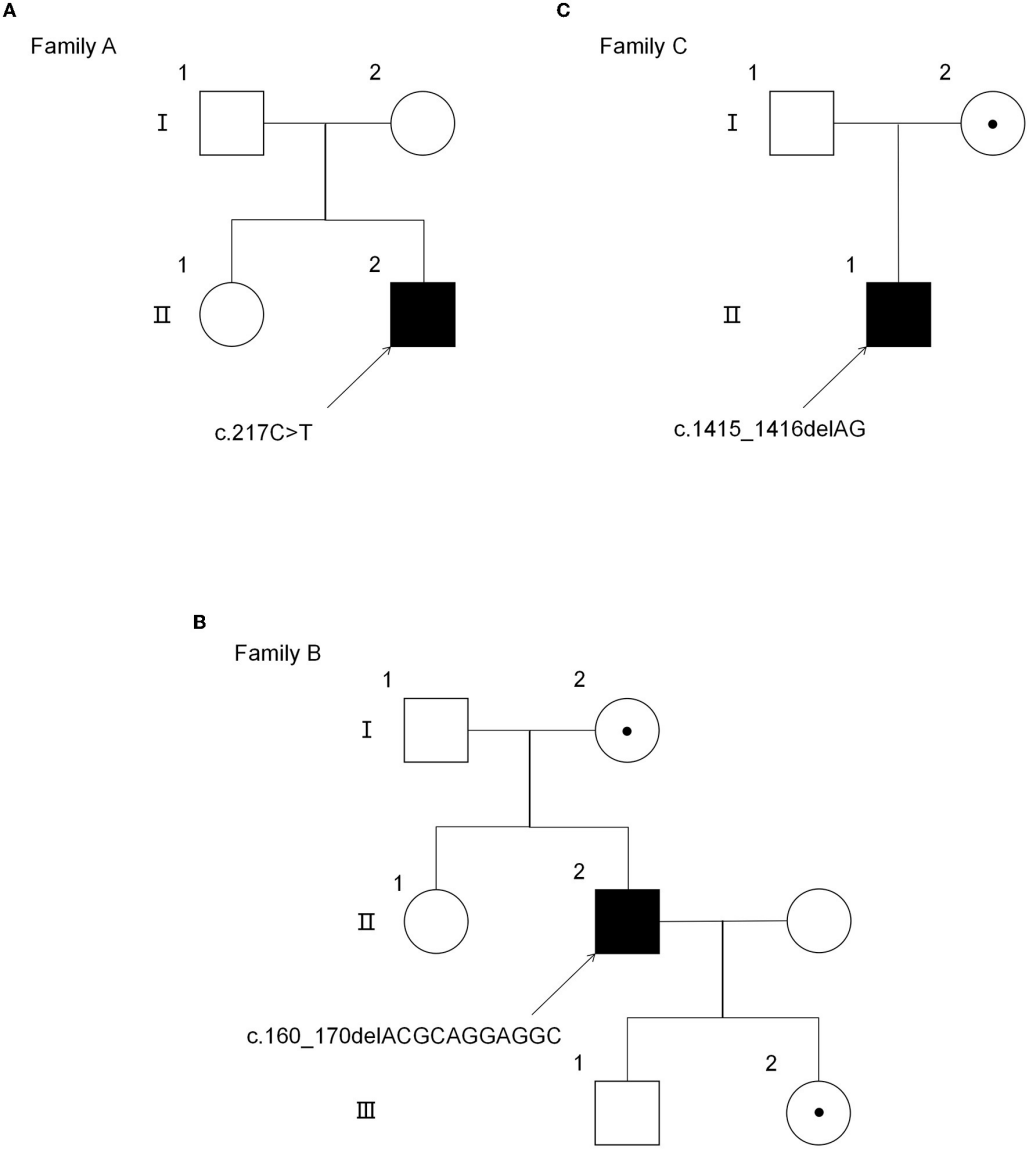
Pedigree of three AMN families. **(A)** The pedigree of the AMN family with the mutation p.Gln73*. **(B)** The pedigree of the AMN family with the mutation p.Thr54Leufs*137. **(C)** The pedigree of the AMN family with the mutation p.Gln472Argfs*83. *Premature stop codon.

Three Chinese Han families were included in our study. Family pedigrees for all the subjects are depicted in Figure 1. All the patients in this study were men (Patient 1: Family A-II2, Patient 2: Family B-II2, and Patient 3: Family C-II1), and their family members had no similar symptoms or signs. The clinical characteristics of the three probands are summarized in Table 1. All three patients were initially suspected of having hereditary spastic paraplegia (HSP). In addition, patient 2 was also initially suspected of familial amyotrophic lateral sclerosis (ALS), because his father was diagnosed with ALS.

**Table 1:** Clinical characteristics of three patients with AMN.

**Patients No**.	**1 (II-2, F1)**	**2 (II-2, F2)**	**3 (II-1, F3)**
Gender	Male	Male	Male
Age at diagnosis, years	32	31	37
Age at onset, years	25	30	34
Initial symptoms	Weakness of lower limbs; spastic gait	Pain in lower limbs; spastic gait	Weakness of lower limbs
Spinal symptoms	Spastic paraparesis	Spastic quadriparesis	Spastic paraparesis
Peripheral neuropathy	Yes	Yes	No
Cognitive impairment	No	No	No
Sphincter dysfunction	No	No	No
Hypoadrenocorticism	Yes (scanty scalp hair, skin pigmentation)	No	No
Muscle strength (UL/ LL)	5/4	3/3	5/4
Muscle tension	Increased	Increased	Increased
Tendon reflexes (UL/LL)	+++/++++	++++/++++	+++/++++
Hoffman sign	Positive	Positive	Positive
Babinski sign	Positive	Positive	Positive
Sensory	Impaired distal pin sensation and vibration sensation	Normal	Normal
Cerebral involvement in MRI	Yes	Yes	Yes
Spinal involvement in MRI	Yes	Yes	No
Disease progression	Slow progression	Rapid progression	Slow progression

M, male; UL, upper limb; LL, lower limb; +, decreased reflex; ++, normal deep reflex; +++, brisk reflex; ++++, hyperreflexia.

The onset of the symptoms in the patients was between 25 and 37 years of age, with the time from onset of symptoms to diagnosis ranging from 1 to 7 years. The initial and major neurological presentations of all three patients were weakness in extremities and gait disorder. In addition, patient 1 suffered from slight numbness of the lower extremities and presented with scanty scalp hair and skin pigmentation.

On neurological examination, all patients presented with significant pyramidal tract signs, including spastic paraparesis or quadriparesis, increased muscle tension and tendon reflexes, and positive Babinski's and Hoffman's signs. Among the three patients, only patient 1 presented with slight impairment of the distal pin-prick sensation and vibration sensation during the sensory examination. However, on electrophysiological examination, patient 1 and patient 2 had mild-to-moderate slowing sensory conduction velocities and reduced sensory nerve action potential amplitudes, which proved the impairment of sensory nerves. Patient 3 had no signs or symptoms of peripheral neuropathy but presented mildly prolonged distal motor latency and reduced motor conduction velocity, with normal sympathetic skin response (SSR). In terms of cognitive function, the scores of the Mini-Mental State Examination (MMSE) and the Montreal Cognitive Scale (MoCA) were normal in all these three patients.

Noteworthy, patient 2 had rapid disease progression within a year after onset. He was unable to walk independently and had mild hearing loss and visual impairment. As the disease progressed, he required constant nursing care and attention as he was now bedridden and incontinent.

### 3.2. Neuroimaging characterization

**Figure 2:**
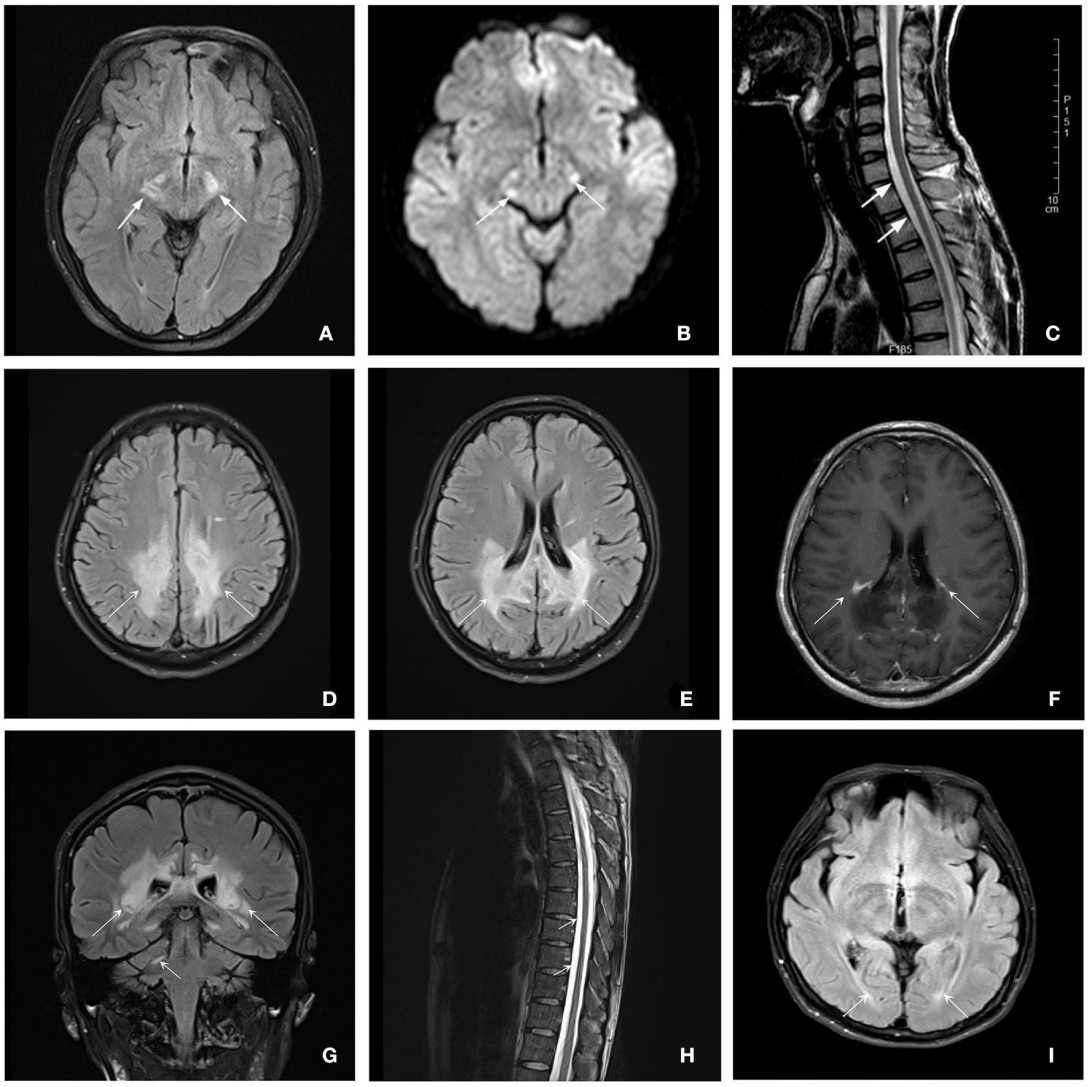
Brain and spinal MRI for patients with ALD. Patient 1 **(A–C)**: **(A, B)** Axial T2-flair and DWI images of brain MRI showed a hyperintensity of bilateral cerebral peduncle (7 years after disease onset) (white arrows). **(C)** Sagittal T2-weighted image of spinal MRI showed the white arrows show lower cervical and upper thoracic spinal cord local atrophy detected (6 years after disease onset) (white arrows). Patient 2 **(D–H)**: **(D, E)** Axial T2-flair images showed extensive hyperintensity involving the frontal and parietal white matter and corpus callosum (white arrows). **(F)** Axial enhanced T1-weighted MRI showed partial linear enhancement at the lesion edge (white arrows). **(G)** Coronal T2-flair images showed extensive hyperintensity involving the periventricular white matter and the right dentate nucleus of the cerebellar (white arrows). **(H)** Sagittal T2-weighted image of spinal MRI indicated the thoracic spinal cord local atrophy detected (white arrows). Patient 3 (i): **(I)** Axial T2-flair and DWI images of brain MRI showed a slight hyperintensity of the periventricular white matter next to posterior horns (3 years after disease onset) (white arrows).

The brain MRI was performed on all patients, and the spinal MRI was performed on two patients (patient 1 and patient 2) (Figure 2). The brain MRI of three patients showed different degrees of white matter lesions involved, including cerebral peduncle, the frontoparietal and periventricular white matter, and corpus callosum. In addition, the brain MRI of patient 2 also showed partial linear enhancement at the lesion edge. Spinal MRI scans of two patients showed thoracic local cord atrophy.

### 3.3. Biochemical characterization

The plasma VLCFAs and serum ACTH levels of the patients were measured, as shown in Table 2. In all three patients, the C26:0 level, and the ratios of C24:0/C22:0 and C26:0/C22:0 increased. The plasma C24:0 level of patient 1 also increased significantly. In addition, the levels of ACTH of two patients (patient 1 and patient 2) were elevated as well but were normal in patient 3.

**Table 2:** Biochemical characteristics of three patients with AMN.

**Biochemical test (reference range)**	**Patient 1**	**Patient 2**	**Patient 3**
C22:0 ( ≤ 96.3 nmol/ml)	64.6	44.4	52.5
C24:0 ( ≤ 91.4 nmol/ml)	108.4	78.6	82.4
C26:0 ( ≤ 1.30 nmol/ml)	4.30	3.03	2.92
C24:0/C22:0 ( ≤ 1.39)	1.68	1.77	1.57
C26:0/C22:0 ( ≤ 0.023)	0.067	0.068	0.056
ACTH (7.2–63.3 pg/ml)	803.2	83.0	62.1

### 3.4. Mutations and bioinformatics analysis

**Figure 3:**
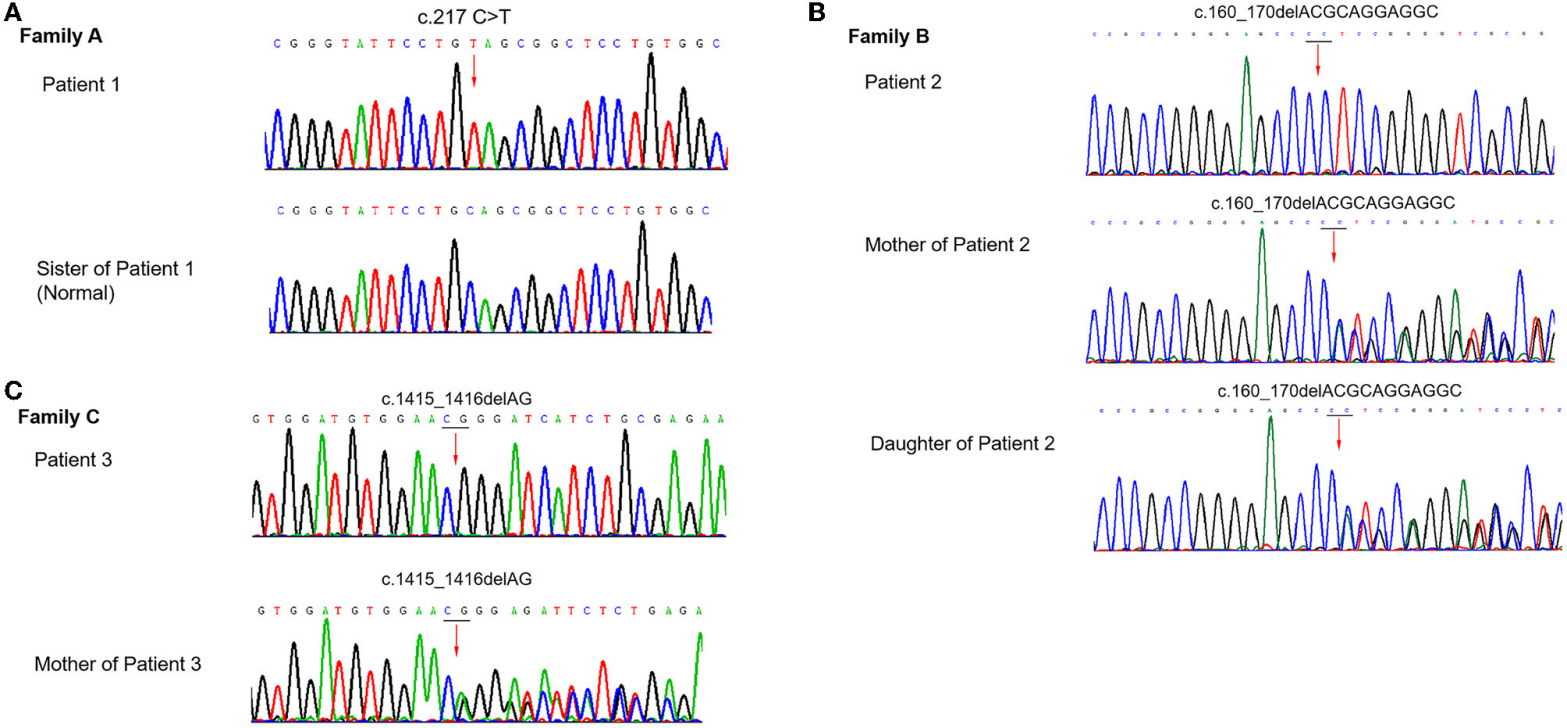
Sanger sequencing results of the ABCD1 gene in the probands and their families. **(A)** Family A: Patient 1 was with mutation c.217 C>T. **(B)** Family B: Patient 2, his mother, and daughter were with the mutation c.160_170delACGCAGGAGGC. **(C)** Family C: Patient 3 and his mother were with the mutation c.1415_1416delAG.

To identify the disease-causing variants, we performed WES for these patients. Three distinct variants were identified and confirmed by Sanger sequencing, as shown in Figure 3.

In the proband (II-1) of family A, a heterozygous nonsense mutation c.217 C>T, p.Gln73^*^ in the ABCD1 gene was detected, which introduced a premature termination codon at the 73rd amino acid of the protein preventing translation of the full-length protein. His mother refused the genetic testing. In family B, the genetic sequencing identified a deletion mutation c.160_170delACGCAGGAGGC in the ABCD1 gene, leading to the frameshift and premature transcription termination of amino acid p.Thr54Leufs^*^137. Sanger sequencing revealed that his mother and daughter carried the hemizygous ABCD1 p.Thr54Leufs^*^137 mutation. In family C, the genetic sequencing showed a deletion mutation c.1415_1416delAG in the ABCD1 gene, leading to the frameshift and premature transcription termination of amino acid p.Gln472Argfs^*^83. His mother also carried the hemizygous ABCD1 p.Gln472Argfs^*^83 mutation.

Among these mutations of the ABCD1 gene, two mutations c.217C>T and c.160_170delACGCAGGAGGC had not been previously in the dbSNP, 1,000 Genomes, gnomAD, or X-ALD database (www.x-ald.nl). The potential pathogenicity of the mutations was investigated by prediction bioinformatics tools mentioned earlier, suggesting all three mutations had the possibility to be disease-causing. According to the variant classification guideline of the American College of Medical Genetics (ACMG), all three mutations could be classified as pathogenic.

**Figure 4:**
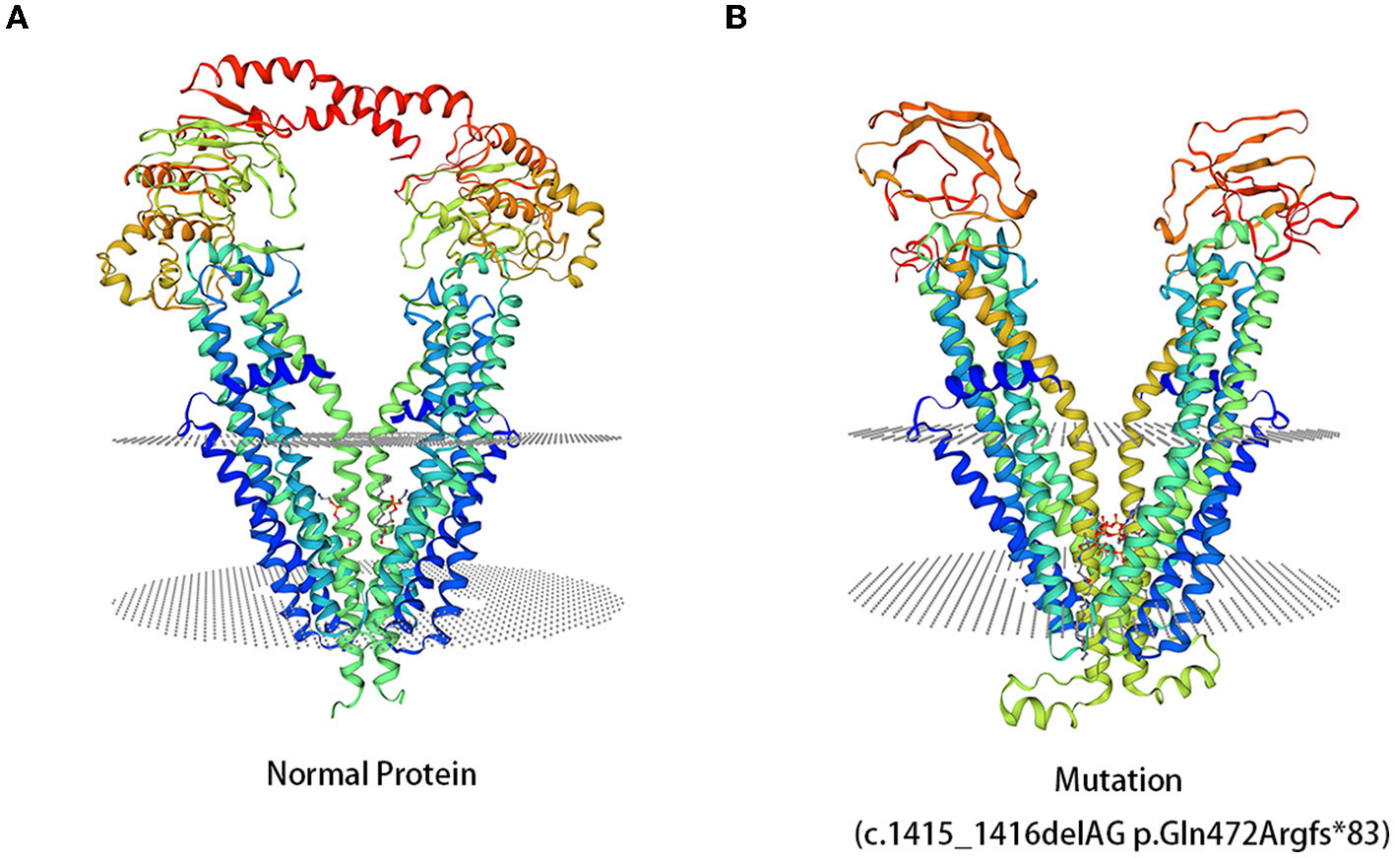
3D computer model (SWISS-MODEL) shows the predicted structures of the ABCD1 protein. **(A)** The predicted structures of the normal ABCD1 protein. **(B)** The predicted structures of the ABCD1 protein carrying the mutation of c.1415_1416delAG p.Gln472Argfr*83.

Compared to the normal structure of the ABCD1 protein, c.1415_1416delAG p.Gln472Argfs^*^83 mutation resulted in different structures of the ABCD1 protein (Figure 4). However, the amino acid sequences of the mutations c.217C>T p.Gln73^*^ and c.160_170delACGCAGGAGGC p.Thr54Leufs^*^137 had low global model quality estimation (GMQE) scores because of the introduction of premature stop codons, which led to unreliable predictions for protein structures.

## 4. Discussion

Adrenoleukodystrophy is a rare X-linked recessive inherited neurodegenerative disorder. AMN is considered the milder default manifestation of X-ALD (9), characterized by slowly progressive non-inflammatory adult-onset spinal cord axonopathy with associated demyelination, peripheral neuropathy, sphincter disturbances, adrenal insufficiency, and hypogonadism (10). Most men with ALD will develop slowly progressive myeloneuropathy in their 20's or 30's (11). Approximately 50% of female carriers may develop AMN manifesting as mild-to-moderate spastic paraparesis in middle-aged women or later with normal adrenal function (1). However, heterogeneity exists in the symptom of onset and severity of symptoms in X-ALD, resulting in difficulties in early diagnosis. In our study, all three AMN probands manifested with symptoms of spastic gait disturbance, without very prominent symptoms of adrenal insufficiency, leading to the initial misdiagnosis of HSP. Patient 1 and patient 3 experienced relatively slow disease progression, while patient 2 had a rapid progression after onset.

Due to Wallerian degeneration, brain MRI demonstrates moderate hyperintensity on FLAIR and T2 sequences of brainstem pyramidal tract, pons, and internal capsule in some patients with AMN but otherwise normal or slightly abnormal brain MRI in most cases like patient 1 and patient 3 (2, 12, 13). However, ~20% of patients with AMN also suffered a rapidly progressive cerebral inflammatory demyelination within the 10 years after disease onset, while presenting cognitive dysfunction (3). Moreover, if the demyelinating lesions progress to the active stage of neuroinflammation, showing the enhancing lesions on brain MRI, the prognosis will be worse, similar to our patient 2. The patient did not show any visual or hearing impairment, cognitive impairment, or obvious psychiatric symptoms when he was diagnosed 5 months after disease onset, even though the brain MRI showed extensive cerebral demyelination involves other than the internal capsule and centrum semiovale, and even the corpus callosum and frontoparietal white matter at this time. He presented with a rapid decline in cortical function characterized by visual and hearing dysfunction at the 9-month follow-up after diagnosis. Patient 1, with relatively slight white matter lesions on brain MRI, did not show any cognitive impairment or mental disorders at the 1-year follow-up after diagnosis. On the other side, this indicates that abnormal white matter imaging may precede clinical signs and symptoms and may be a potential predictor of progression, which is in accordance with previous studies (14, 15). However, spinal cord atrophy seemed not to be necessarily consistent with the severity of spastic paralysis.

The increased level of VLCFA in plasma is the principal biochemical abnormality in ALD. The accumulation of VLCFA in white matter and the adrenal cortex leads to clinical and imaging abnormalities in patients with ALD; however, it is irrelevant to the clinical signs and the severity of neuroimaging according to the previous research and our results (2, 16). For the relationship between the change in VLCFA level and disease progression, there is currently a lack of further clinical evidence. Another important biochemical feature of ALD is the increased level of ACTH, although its sensitivity for the diagnosis of ALD is lower than VLCFA. Previous research has found that more than 33% of patients with AMN have normal ACTH levels (2, 15). The ACTH level of two patients in our study increased, but only patient 1 had mild signs of adrenal insufficiency.

The symptoms of X-ALD are complex and non-specific, which can easily result in misdiagnosis and missed diagnosis, as was the case in our study. All patients were suspected of HSP before AMN diagnosis. Thus, gene diagnosis is one of the most important criteria for the diagnosis. Given that some patients had a positive family history of other neurodegenerative diseases or lacked HSP-related mutations, additional WES and Sanger sequencing verification was warranted in our study, which played a decisive role in the genetic analysis. To date, over 960 different mutations in X-ALD have been reported (www.x-ald.nl). In the present study, we identified one nonsense and two frameshift mutations in the ABCD1 gene in three AMN families. Of the three mutations, c.1415_1416delAG in exon5 is one of the most commonly recurring pathogenic variants of the ABCD1 gene, which can lead to the truncation and negative expression of the adrenoleukodystrophy protein (ALDP). Most cases occurred in newborns and adolescents and appeared as cerebral ALD (CALD) phenotype (17, 18). Though this mutation is comparatively rarely reported in patients with late-onset spastic paraplegia as the first symptom, our results are consistent with the existing reports on AMN (19, 20). Patient 2 with c.160_170delACGCAGGAGGC suffered from behavioral and personality changes, visual impairment, and auditory processing problems within a short course of clinical onset, except for rapidly progressive spinal cord symptoms. Compared with the truncated protein caused by c.1416_1417del, the mutations (c.217C>T and c.160_170delACGCAGGAGGC) were expected to produce severely truncated protein, but they do not appear to have the same effect on disease severity and progression in AMN. This means that there is no genotype–phenotype correlation, even no clear correlation between disease severity and genotype, as shown in previous studies (21, 22). This is because, in addition to ABCD1 gene mutations, other triggers such as trauma, genetics, epigenetics, and environmental factors may also be involved in the pathogenesis of ALD (2). Thus, the relationship between genotype and clinical phenotype will require further evaluation in future studies.

As we know, leukodystrophies are only rarely considered in the differential diagnosis of progressive spastic paraplegia, especially in adult-onset cases. A study population mainly of European origin and a Chinese study showed that leukodystrophies were ~11–21% of cryptic, adult-onset, lower limb spasticity with ALD being the most frequent cause (5–6%). These proportions seemed relatively high in the Chinese population compared with the European population (23, 24). However, due to the heterogeneous presentation of adult-onset X-ALD, diagnosis is often challenging. Clinical presentation and genetic detection play a major role in diagnosing X-ALD, supplemented with VLCFA examination and neuroimaging. Although diagnostic approaches are known to have remarkably improved in recent decades, both misdiagnosis and underdiagnosis are common due to the limited understanding of X-ALD. Our results supported that X-ALD is an important cause of spastic paraparesis and ABCD1 gene should be included in the genetic sequencing, especially for late-onset spastic paraplegia patients with leukoencephalopathy, peripheral neuropathy, cognitive impairment, or adrenocortical insufficiency.

Furthermore, advances in therapeutic options for ALD also make early diagnosis important. For instance, a hematopoietic stem cell transplant (HSCT) is able to halt the progression of CALD, improving survival and function, especially in early CALD (25), but despite the gratifying results from HSCT for CALD, its efficacy on AMN is unclear (11). Previous studies have pointed out that HSCT for CALD in childhood does not prevent the development of AMN in adulthood (26). Meanwhile, some scientists began to explore gene-based treatment options for AMN. Several studies have successfully transduced CNS cells *in vitro* with adenoviral vectors containing human ABCD1 and proved that ABCD1 protein is localized in peroxisomes, with a reduction in VLCFAs (27, 28). Thus, gene therapy for AMN is worth looking forward to clinical translation.

In conclusion, we reported three X-linked inherited families with adult-onset AMN and identified two novel mutations (p.Gln73^*^ and p.Thr54Leufs^*^137) in the ABCD1 gene. These three cases were all initially considered as HSP and finally diagnosed with AMN by genetic, biochemical, and imaging testing. In conclusion, the variable manifestations of AMN mean that individual patients with AMN may have different, partially overlapping combinations of clinical symptoms and signs. This study also highlights that timely and correct genetic testing strategies are important for avoiding unnecessary diagnostic procedures for rare genetic diseases, such as X-ALD.

## Data availability statement

The original contributions presented in the study are publicly available. This data can be found here: https://www.ncbi.nlm.nih.gov/sra, accession number: PRJNA931945 and https://www.ncbi.nlm.nih.gov/biosample, accession numbers: SAMN33319136, SAMN33319137, and SAMN33319138.

## Ethics statement

The studies involving human participants were reviewed and approved by Ethics Committee of Fujian Medical University Union Hospital. The patients/participants provided their written informed consent to participate in this study. Written informed consent was obtained from the individual(s) for the publication of any potentially identifiable images or data included in this article.

## Author contributions

RH designed the study, collected and analyzed the data, interpreted the results, and wrote the manuscript. JZ designed the study and collected the data. TH and GC supervised the study. QY and ZZ designed the study, supervised the study, and revised the manuscript. All authors contributed to the article and approved the submitted version.
